# Electroacupuncture alleviates ulcerative colitis by targeting CXCL1: evidence from the transcriptome and validation

**DOI:** 10.3389/fimmu.2023.1187574

**Published:** 2023-09-01

**Authors:** Rui-Bin Zhang, Long-Cong Dong, Yuan Shen, Hong-Ying Li, Qin Huang, Shu-Guang Yu, Qiao-Feng Wu

**Affiliations:** Acupuncture and Tuina College, Chengdu University of Traditional Chinese Medicine, Chengdu, China

**Keywords:** ulcerative colitis, electroacupuncture, transcriptome, CXC motif chemokine ligand 1, immune infiltrating cells, Th1 cytokine interferon-γ, macrophages

## Abstract

**Background:**

We aimed to use transcriptomics, bioinformatics analysis, and core gene validation to identify the core gene and potential mechanisms for electroacupuncture (EA) treatment of ulcerative colitis (UC).

**Materials and methods:**

EA was performed in mice after induction of UC *via* dextran sodium sulfate. Body weight, disease activity index (DAI), colon length, and hematoxylin-eosin of the colon tissue were used to evaluate the effects of EA. Mice transcriptome samples were analyzed to identify the core genes, and further verified with human transcriptome database; the ImmuCellAI database was used to analyze the relationship between the core gene and immune infiltrating cells (IICs); and immunofluorescence was used to verify the results.

**Results:**

EA could reduce DAI and histological colitis scores, increase bodyweight and colon length, and improve the expression of local and systemic proinflammatory factors in the serum and colon of UC mice. Eighteen co-differentially expressed genes were identified by joint bioinformatics analyses of mouse and human transcriptional data; *Cxcl1* was the core gene. EA affected IICs by inhibiting *Cxcl1* expression and regulated the polarization of macrophages by affecting the Th1 cytokine IFN-γ, inhibiting the expression of CXCL1.

**Conclusions:**

CXCL1 is the target of EA, which is associated with the underlying immune mechanism related to Th1 cytokine IFN-γ.

## Introduction

Ulcerative colitis (UC) is an inflammatory condition of the colon and rectum that is chronic, refractory, and relapsing (1). The major symptoms of UC are abdominal pain, bloody mucopurulent stool, and diarrhea (2). In addition, UC reduces quality of life and contributes to a shortage of medical resources, resulting in a large medical and socioeconomic burden on society (3, 4). Regrettably, UC has had increased incidence in recent years and remains a global health issue (5).

Currently, effective therapy for UC is limited. Although 5-Aminosalicylic Acid (5-ASA) drugs (including sulfasalazine, mesalamine, and diazo-bound 5-ASA) are commonly used to treat UC, these drugs have unexpected side effects (6, 7). Electroacupuncture (EA), as a Chinese medicine therapy, is widely used to treat UC, with good curative effects (8). Studies have shown that EA has anti-inflammatory or immunomodulatory effects on UC (9, 10). Despite this, the anti-inflammatory properties and effect of EA on the immune cells has not been well studied, and the critical target genes of EA in the treatment of UC remain unclear.

Transcriptome sequencing can capture the dynamic changes of transcription in different tissues and pathophysiological states to further reveal associations between significant differentially expressed genes and biological functions (11). The genetic similarity between human and mice makes mice a popular model to study human diseases (12). Consequently, analyzing the colonic transcriptomes of UC mice after EA interventions will help to determine the effective mechanism and core genes (13). In order to mitigate the potential for biased outcomes stemming from animal experimentation, it is imperative to undertake cross-species integration of transcriptome data from both mice and humans as a means of investigating the underlying mechanisms of EA. (14).

UC is a pathological condition that arises from aberrant immune responses, which can be either innate or adaptive in nature (15). This is mainly manifested as an outbreak of pro-inflammatory cytokines, such as IFN-γ, IL-1β, TNF-α and IL-6. There are several immune cells involved in releasing pro-inflammatory cytokines, and macrophages polarization play a crucial role during the acute phase of colitis. (16). Macrophages can be divided into classic activated type (M1 type) and alternative activated type (M2 type) after being changed by the surrounding microenvironment. IFN-γ can induce typical activation of macrophages (M1 type), which secrete pro-inflammatory cytokines and cause mucosal damage (17). However, it is still unclear whether electroacupuncture can regulate macrophage polarization in UC.

We aimed to identify the core genes regulated by EA through transcriptome data analysis of mice and humans. Furthermore, immune-infiltrated cells (IICs) related to UC were screened by the ImmuCellAI database, and the correlation between IICs and the core gene was calculated to identify immune-related gene regulated by EA. In the treatment of UC with EA, the comprehensive analysis of IICs and core genes reveals relevant biomarkers and potential therapeutic mechanisms.

## Materials and methods

### Experimental animals and groups

All animal experiments were performed according to the animal protocols approved by the Animal Experimental Ethics Committee of the Chengdu University of Traditional Chinese Medicine (2019-04). C57BL/6J mice (SPF class, 26 ± 2 g) were provided by GemPharmatech (Nanjing, China). All mice were housed in a specific pathogen-free environment and subjected to 12 hours of light/dark cycles under ambient temperatures of 23 ± 2°C. We divided the mice into a control, dextran sodium sulfate (DSS), and DSS combined with the EA (DSS + EA) groups.

In the DSS and DSS + EA groups, mice were given 2.5% DSS (MP Biomedicals, California, USA) in drinking water for 7 days (18); whereas the control group received distilled water. Daily body weight and fecal bleeding were evaluated in each group.

### EA intervention

After 5 days of UC modeling, mice in the DSS + EA group were treated with EA once a day for 5 days (19). A custom-made mouse frame was used to hold the animals and both of the Zusanli acupoint points (ST36, located ~3 mm below the capitulum fibulae) were targeted by EA for 30 minutes (2 Hz, 0.2 mA) (20), the hind limbs of the mice began to tremble slightly. Similarly, the control and the DSS groups were held for 30 minutes without EA intervention.

### Disease activity index scoring

The DAI was calculated using the following formula: DAI = (weight loss [%] + represented stool + blood in stool)/3. The details are included in **Supplementary Table 1**.

### Hematoxylin-eosin staining

The mice were killed after 5 days of treatment, and the colon sections were processed for HE staining. As shown in **Supplementary Table 2**, inflammation extent and crypt damage were combined into a histological score. The sections were scanned with an HS6 digital total microtome (Sunny, Shanghai, China).

### Enzyme-linked immunosorbent assay

Whole blood was collected and allowed to clot for 15 min at room temperature to obtain serum. Centrifugation at 3000 rpm for 20 minutes was used to collect serum from the different groups. As directed by the manufacturer, ELISA kits (Elabscience, Wuhan, China) were used to detect tumor necrosis factor TNF-α and IFN-γ levels in the supernatant. Following that, absorption coefficients were applied to calculate TNF-α and IFN-γ concentrations.

### Western blot

Protease and phosphatase inhibitors were added to RIPA lysis buffer to lyse the colon tissue proteins. Bicinchoninic acid (BCA) protein assay kit (Thermo, Rockford, USA) was used to quantify protein concentrations. The protein extracts (30 μg) from the colon tissue were loaded in FuturePAGETM gel (ACE, ET15420GEL, Nanjing, China) and transferred onto PVDF membranes. The membranes were then blocked with 5% non-fat dry milk in Tris-buffered saline/Tween 20 (TBST) and incubated with primary antibodies (anti-IL6, anti-IL1β, anti-TNF-α, anti-IFN-γ, anti-CXCL1, anti-GAPDH, anti-actin) overnight at 4°C. The secondary antibody was then incubated for an additional 2 hours. WB antibody was then performed and the concentrations of the following were determined: anti-IL6 (Bioss, bs-0782R, 1:1000); anti-IL1β (Bioss, bs-0812R, 1:500); anti-TNF-α (Proteintech, 60291-1-Ig, 1:1000); anti-IFN-γ (Bioss, bs-0480R, 1:1000); anti-CXCL1 (Proteintech, 12335-1-AP, 1:1000); anti-GAPDH (Proteintech, 60004-1-Ig, 1:5000); and anti-actin (Proteintech, 66009-1-Ig, 1:5000).

### Mice transcriptome data collection and processing

A total RNA sample was extracted from the colon tissue from mice in each experimental group (three mice per group). An Oxford Nanopore Technology (ONT, Oxford, UK) protocol was followed to generate the cDNA library. The sequencing was conducted by Biomarker Technologies Corp (Beijing, China). Additionally, this ONT data has been deposited into the GEO database (http://www.ncbi.nlm.nih.gov/geo), Accession number: GSE227407.

### Human transcriptome data collection

Data from human colon biopsies was downloaded from the GEO database (http://www.ncbi.nlm.nih.gov/geo). We downloaded expression files GSE38713 for UC, including 28 samples as follows: 13 samples from normal subjects (Normal group) and 15 samples from UC patients (UC group).

### Differentially expressed genes screening

Genes exhibiting |log2 (fold change)| >2.5 and P-value < 0.01 were defined as the DEGs. The DEGs were analyzed using DESeq2 R software package (3.6.3) on mice and human transcriptome data (21).

### Biological function enrichment analysis

The gene ontology (GO) annotations were divided into the following sections: biological processes (BP); molecular functions (MF); and cellular components (CC). Kyoto Encyclopedia of Genes and Genomes (KEGG) pathway enrichment analyses were performed by DAVID (https://david.ncifcrf.gov/) (22). Visualization of the enrichment results were achieved using the R ggplot2 package and Cytoscape V3.7.1 (23).

### Protein-protein interaction network

Homologous gene mapping was performed using the STRING database (http://string-db.org) (24), and PPI network was constructed. Cytoscape Version 3.7.1 was then used to visualize and analyze the protein interactions.

### Quantitative real-time polymerase chain reaction

Total RNA was isolated from the colon tissues using a MolPure^®^ TRIeasyTM Plus Total RNA Kit (Yeasen, Shanghai, China). Hifair^®^III 1st Strand cDNA Synthesis SuperMix (Yeasen, Shanghai, China) was used to prepare the cDNA. We used a Bio-Rad CFX Maestro PCR system for real-time PCR detection with the Hieff^®^ qPCR SYBR^®^Green Master Mix (Yeasen, Shanghai, China). The 2^-△△Ct^ method was used to calculate relative mRNA expression of target genes, and β-actin expression levels were used as endogenous controls. **Supplementary Table 3** lists the primer sequences.

### Immune cell infiltration profile

To elucidate immune cell infiltration in the human samples, we used the Immune Cell Abundance Identifier (ImmuCellAI, http://bioinfo.life.hust.edu.cn/web/ImmuCellAI/) (25), and 24 immune cells were scored for infiltration. Then, the ComplexHeatmap package (26) was applied to draw a heatmap of the IICs. Pearson’s correlation coefficient was used to analyze the correlation between CXC motif chemokine ligand 1 (*Cxcl1*) and IICs.

### Immunofluorescence

The sections of samples were deparaffinized in xylene and rehydrated in decreasing concentrations of alcohol. After washing the sections three times in phosphate buffered saline (PBS) for 5 minutes, we blotted them with 5% goat serum for 1 hour at 37°C. The sections were incubated with the primary antibody overnight at 4°C. These sections were washed three times in PBS and then incubated with secondary antibodies for 2 hours at 37°C. The sections were subsequently rinsed three more times and stained with DAPI (Beyotime Biotechnology, Shanghai, China) for 5 minutes. Pannoramic 250FLASH (3DHISTECH, Budapest, Hungary) was used to image the results. The IF antibody and its concentration were determined for the following: anti-CXCL1 (Proteintech, 12335-1-AP, 1:100); anti-F4/80 (Servicebio, GB11027, 1:500); anti-CD86 (CST, #91882S, 1:200); anti-ARG1 (Servicebio, GB11285, 1:200); anti-CD4 (Servicebio, GB13064-2, 1:200); and anti-IFN-γ (Bioss, bs-0480R, 1:100).

### Statistical analysis

Statistical analysis was performed using SPSS software (version 26.0) and pictures were drawn using GraphPad Prism software (version 8.0). Data are presented as mean ± SEM. The one-way analysis of variance (ANOVA) made multiple-group comparisons. *P*<0.05 was considered statistically significant.

## Results

### EA can alleviate DSS-induced mouse colitis

**Figure 1:**
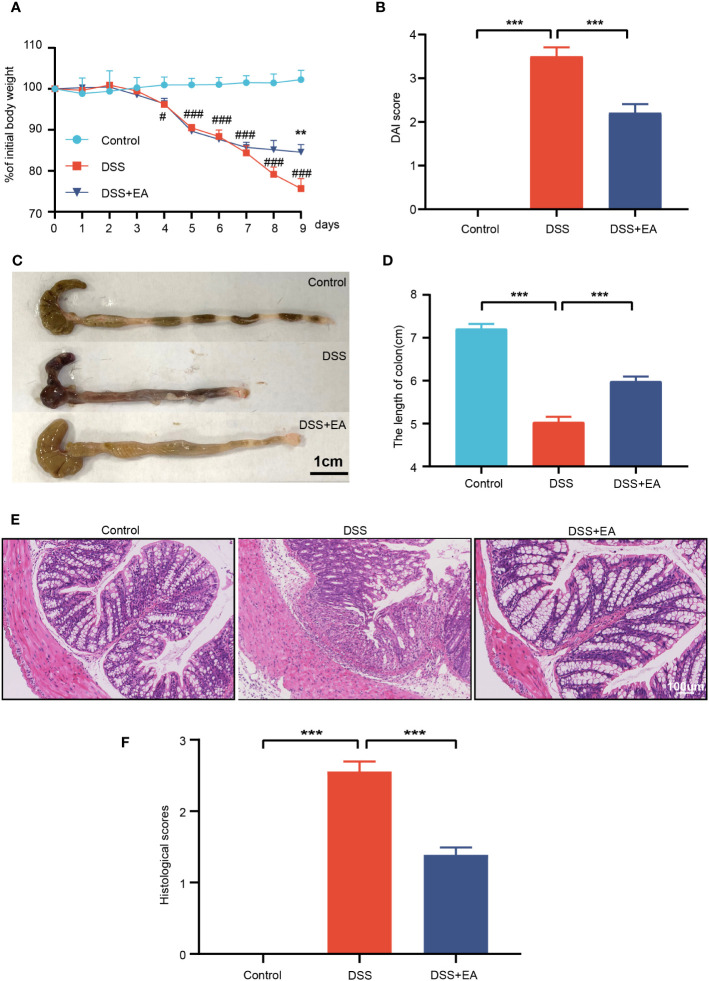
Electroacupuncture of ST36 can relieve ulcerative colitis (UC). **(A)** Percentage of initial body weight (n = 8). **(B)** DAI score (n = 8). **(C)** Representative image of the colon length. **(D)** Colon length (n = 6). **(E)** Representative images of HE staining in the colon tissues (magnification ×200). **(F)** Histological score (n = 6). Data are show as mean ± SEM. **P < 0.01, ***P < 0.001, #*P*<0.05, ###*P*<0.001.

In this study, we first investigated the effect of EA on DSS-induced colitis. Similar to human UC, DSS-treated model mice showed distinct clinical features of UC. Compared with the control group, the body weight of mice in the DSS group decreased significantly but increased significantly after EA treatment (**Figure 1A**). The DAI score of the DSS group was significantly increased compared with the control group but decreased after EA treatment (**Figure 1B**).

In DSS-induced colitis, shortening or atrophy of the colon is an important indicator. Compared with the control group, the colon length of the DSS group decreased significantly, but increased after EA treatment (**Figures 1C, D**). Then, the colon tissue was stained with HE to assess the severity of colitis. Compared with the control group, DSS mice showed overt necrosis, edema, and disappearance of crypt structures in the intestinal mucosa, as well as diffuse infiltration of the inflammatory cells. In contrast, the DSS + EA group had preserved colon morphology and reduced levels of inflammation (**Figures 1E, F**).

### EA can reduce colonic and systemic inflammation in DSS mice

**Figure 2:**
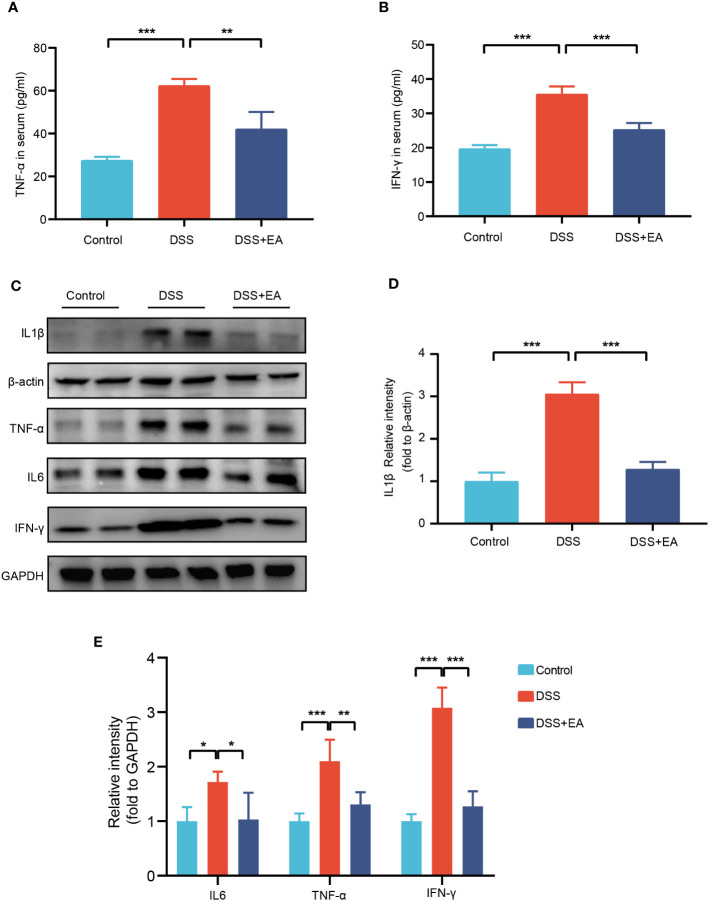
EA of ST36 inhibited systemic and colonic inflammation in DSS mice. **(A)** Serum levels of inflammatory cytokines TNF-α (n = 8). **(B)** Serum levels of inflammatory cytokines IFN-γ (n = 8). **(C)** Western blotting of IL-1β, IL-6, TNF-α, and IFN-γ expressions in colon tissues. **(D)** The relative intensities of IL-1β as normalized against β-actin (n = 4). **(E)** The relative intensities of IL-6, TNF-α, and IFN-γ as normalized against GAPDH (n = 4). Data are show as means ± SEM. *P < 0.05, **P < 0.01, ***P < 0.001. To fit into the manuscript properly, the gel was reasonably trimmed.

Because inflammatory cytokines play an important role in the response to colitis, we detected the expression levels of proinflammatory cytokines TNF-α and IFN-γ in the serum by ELISA. As shown in **Figures 2A, B**, compared with the control group, the serum levels of these cytokines were significantly up regulated in the DSS group, while the levels of these cytokines were significantly inhibited by EA treatment. Meanwhile, through the WB experiment, we found that the expressions of IL1β, IL-6, TNF-α, and IFN-γ in the colon tissues of the DSS group were significantly higher than those of the control group. In contrast, EA treatment significantly reduced DSS-induced upregulation of these inflammatory cytokines (**Figures 2C–E**). In conclusion, these results indicate that EA has an effect on inhibiting systemic inflammation and colonic inflammation.

### Analysis of mice transcriptome samples

**Figure 3:**
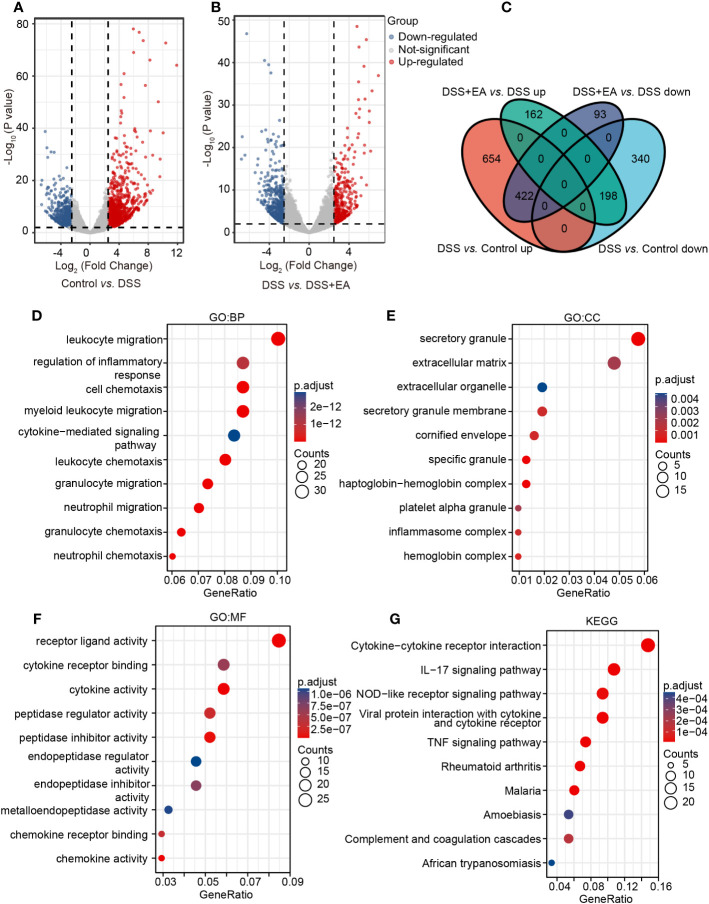
Analysis of mice transcriptome samples. **(A)** Volcano plot for DEGs in the DSS group *vs.* the control group (|log2FC| > 2.5, P < 0.01). **(B)** Volcano plot for DEGs in the DSS + EA group *vs.* the DSS group (|log2FC| > 2.5, P < 0.01). **(C)** Venn diagram of DEGs. **(D)** GO: BP terms of mice DEGs. **(E)** GO: CC terms of mice DEGs. **(F)** GO: MF terms of mice DEGs. **(G)** KEGG pathways of mice DEGs.

To further explore the effective mechanism of EA, mice transcriptome analysis was carried out. A volcano map of mice RNA-seq results showed that, in Control *vs.* DSS, DSS group exhibited 1614 DEGs compared with the control group, with 1076 up-regulated DEGs and 538 down-regulated DEGs (**Figure 3A**). In DSS *vs.* DSS + EA, there were also 875 DEGs between the DSS + EA and DSS groups, of which 360 were up-regulated and 515 were down-regulated (**Figure 3B**). Next, we used Venn diagram to take the intersection of DEGs screened by Control *vs.* DSS and DSS *vs.* DSS + EA. 620 DEGs were found in the intersection set which we defined as mice EA-DEGs (**Figure 3C**).

Next, mice EA-DEGs were analyzed by GO and KEGG pathway enrichment. According to the GO database, three different ontologies were used to annotate the genes, including BP, CC, and MF. From each, we extracted the top 10 enriched terms. In GO BP terms, the mice EA-DEGs were mostly enriched in ‘leukocyte migration’ and ‘regulation of the inflammatory response’ (**Figure 3D**). In GO CC terms, the mice EA-DEGs were mostly enriched in ‘secretory granule’ and ‘extracellular matrix’ (**Figure 3E**). In GO MF terms, the mice EA-DEGs were mostly enriched in ‘receptor ligand activity’ and ‘cytokine receptor binding’ (**Figure 3F**). Based on KEGG pathway analysis, we found that EA regulated the top three pathways, including ‘cytokine-cytokine receptor interaction,’ ‘IL-17 signaling pathway,’ and ‘NOD-like receptor signaling pathway’ (**Figure 3G**).

### Analysis of human transcriptome samples

**Figure 4:**
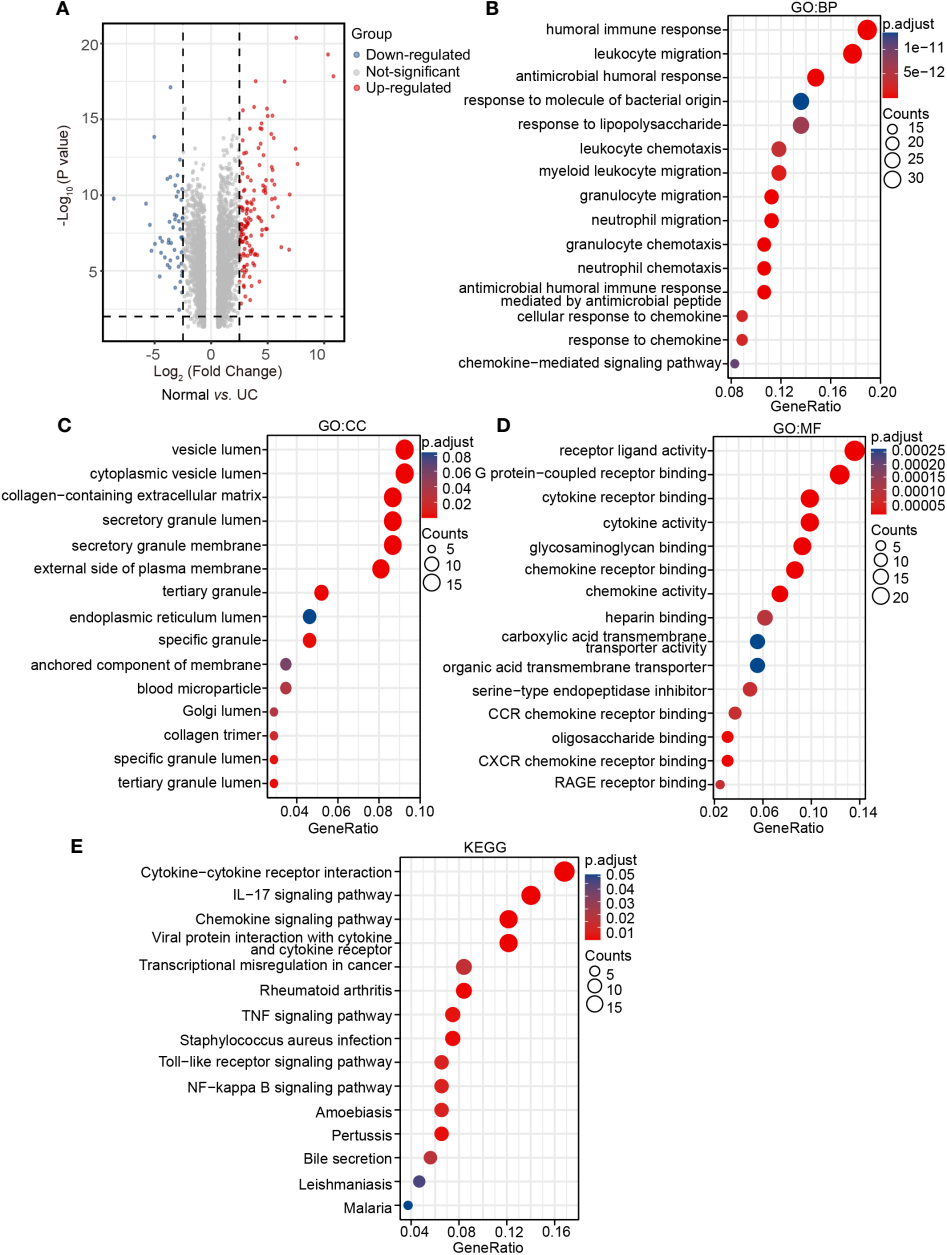
Analysis of human transcriptome samples. **(A)** Volcano plot for human DEGs in the UC group *vs*. the normal group (|log2FC| > 2.5, P < 0.01), **(B)** GO: BP terms of human DEGs. **(C)** GO: CC terms of human DEGs. **(D)** GO: MF terms of human DEGs. **(E)** KEGG pathways of human DEGs.

To fully understand the transcriptional changes caused by the disease, 28 human samples from GSE38713 of the GEO database were analyzed to further verified the results of mice experiments. In the UC patient group, 137 DEGs were up-regulated and 49 DEGs were down-regulated compared with the normal subjects’ group (**Figure 4A**). We defined these 186 DEGs as human DEGs. In GO BP terms, the human DEGs were mostly enriched in ‘humoral immune response’ and ‘leukocyte migration’ (**Figure 4B**). In GO CC terms, the human DEGs were mostly enriched in ‘vesicle lumen’ and ‘cytoplasmic vesicle lumen’ (**Figure 4C**). In GO MF terms, the human DEGs were mostly enriched in ‘receptor ligand activity’ (**Figure 4D**). Many results were similar to the transcription data collected from mice. In the same way, KEGG showed that ‘cytokine–cytokine receptor interaction’ and ‘IL-17 signaling pathway’ were two of the most significant changes in the human samples, which is consistent with our data (**Figure 4E**).

### Integrating mice and human DEGs

**Figure 5:**
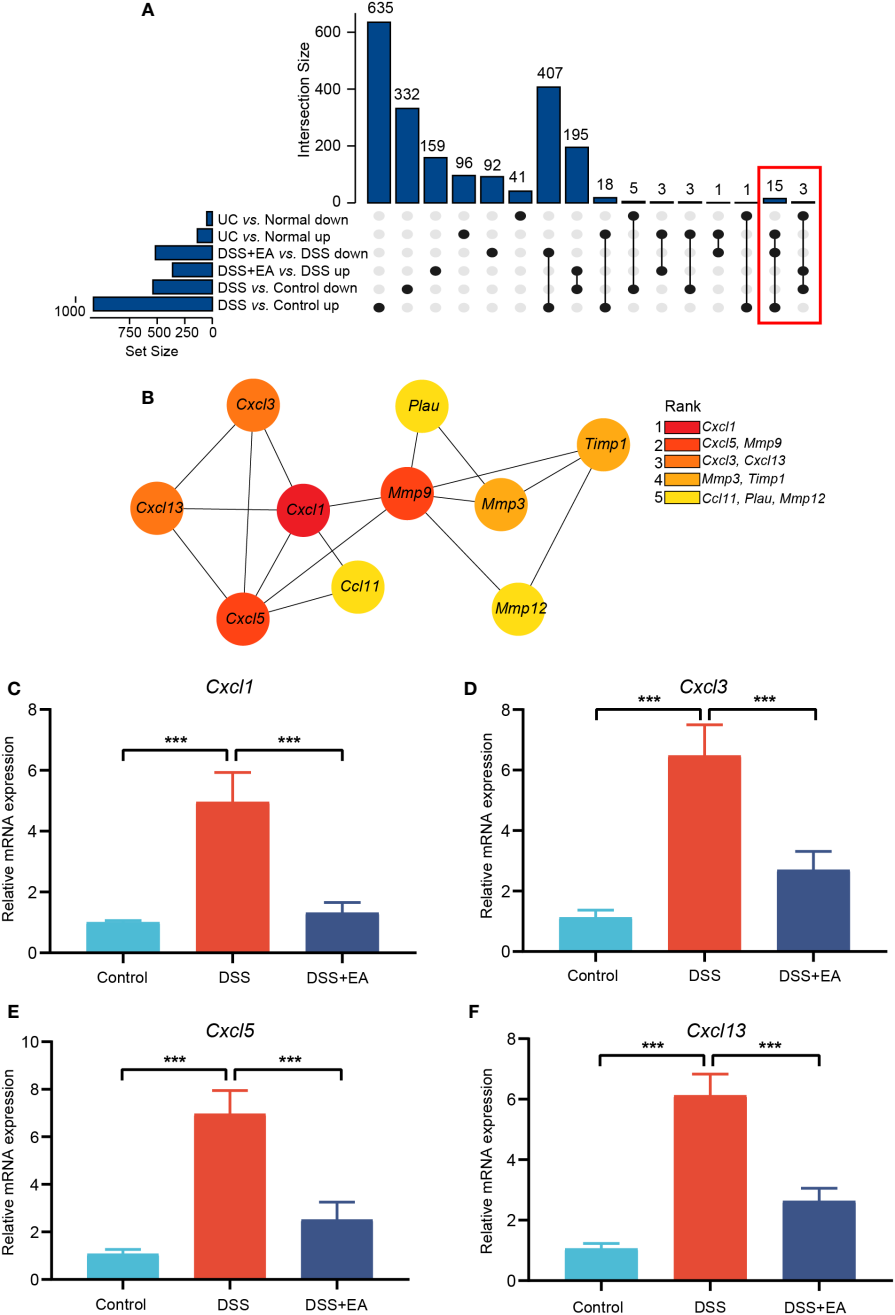
Integrating mice and human DEGs. **(A)** UpSet plot of co-DEGs. **(B)** Subnetwork of co-DEGs identified in STRING. Co-DEGs identified by cytoHubba. Red-yellow color gradient reflects (from high to low) each node’s connection degree. **(C)** Relative expression of *Cxcl1* mRNA (n = 6). **(D)** Relative expression of *Cxcl3* mRNA (n = 6). **(E)** Relative expression of *Cxcl5* mRNA (n = 6). **(F)** Relative expression of *Cxcl13* mRNA (n = 6). Data are show as mean ± SEM. ***P < 0.001.

To further clarify which genes were regulated by EA in mice and those that show high differences in human UC, we integrated mice EA-DEGs and human DEGs. The STRING database was used for homologous gene transformation, and we found that 18 co-differentially expressed genes (co-DEGs) between mice EA-DEGs and human DEGs. This finding suggests that the 18 co-DEGs genes play a crucial role not only in the regulation of DSS mice by EA, but also in the pathogenesis of human UC disease. (**Figure 5A**). Then, based on the identified co-DEGs, a PPI network was constructed using the STRING database. We found that these co-DEGs were mainly divided into two categories: Matrix metalloproteinase (*Mmp9*, *Mmp12*, and *Mmp3*); and the CXC family of chemokines (*Cxcl1*, *Cxcl3*, *Cxcl5*, and *Cxcl13*) (**Figure 5B**).

We used cytoHubba, a plugin of Cytoscape, to find the core gene regulated by EA. With the MCC algorithm, *Cxcl1* was identified as the core gene (**Figure 5B**). Finally, it was found that EA had a significant inhibitory effect on these CXC family chemokines mRNA expression compared to the DSS group by qRT-PCR validation (**Figures 5C–F**).

### Profile of immune infiltration in UC

**Figure 6:**
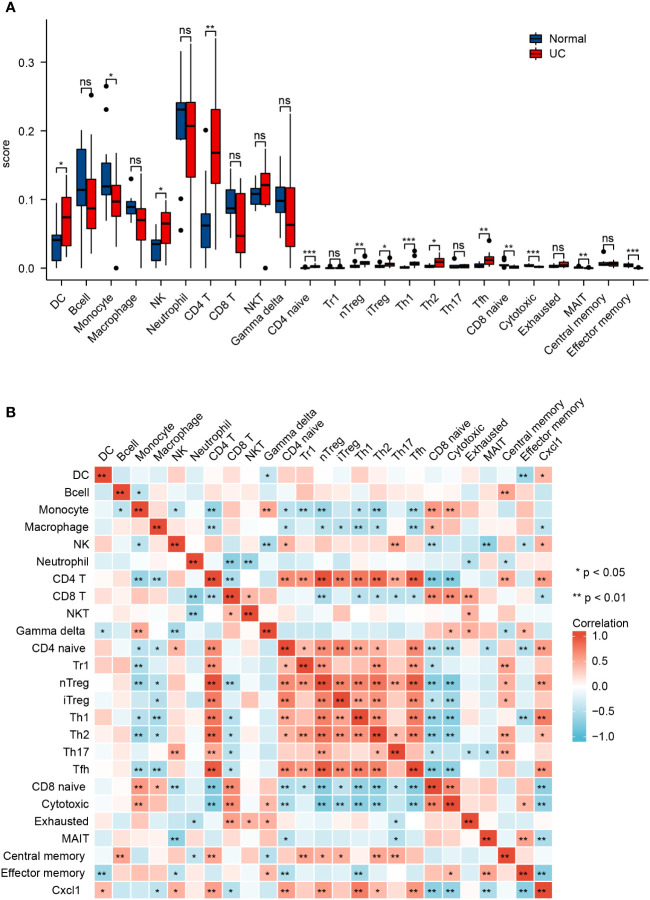
Profile of immune infiltration in UC. **(A)** Violin plot for the different proportions of IICs between the UC group and the normal group. **(B)** Correlation heatmap between IICs and Cxcl1 in the UC group. Data are show as mean ± SEM. *P < 0.05, **P < 0.01, ***P < 0.001.

By using the ImmuCellAI database, we analyzed immune infiltrations of GSE38713. As shown in **Figure 6A**, the fraction of DC, NK, CD4+ T, CD4 naïve T, nTreg, iTreg, Th1, Th2, and Tfh cells in the UC group had higher levels than the normal group; however, the levels of monocytes, CD8 naïve cells, cytotoxic cells, MAIT, and effector memory cells were lower. We then found that *Cxcl1* expression levels were closely correlated with Th1, Tfh, CD4+ T, nTreg, and CD4 naïve T cells (Pearson’s correlation coefficient > 0.5, P < 0.01) (**Figure 6B**). These results further indicate that *Cxcl1* is strongly associated with IICs. The results suggest that EA may affect IICs by inhibiting the expression of *Cxcl1*, thereby treating UC.

### EA can regulate the polarization of macrophages by affecting the Th1 cytokine IFN-γ, thereby inhibiting the expression of CXCL1

**Figure 7:**
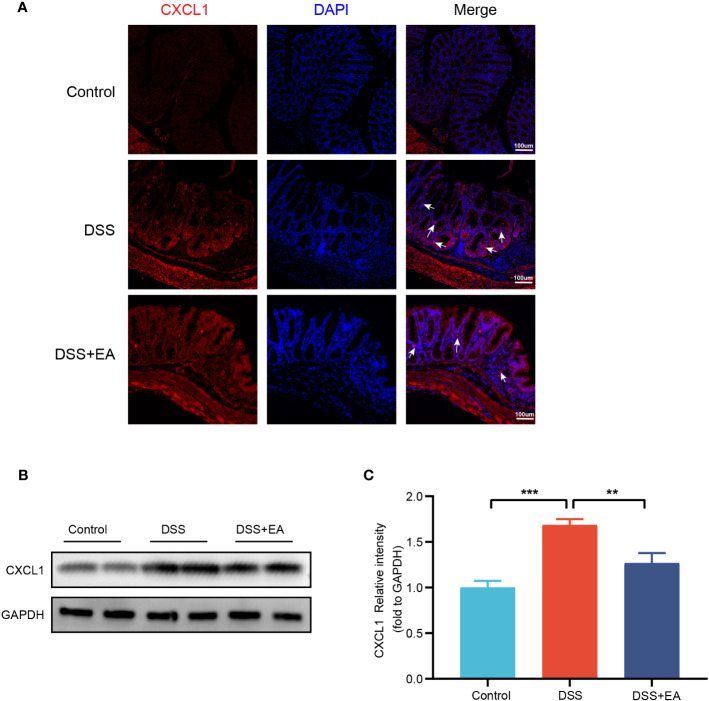
Colonic CXCL1 expression in the different groups. **(A)** CXCL1 immunohistochemical staining (red expresses CXCL1-positive cells and blue expresses DAPI). **(B)** Western blot of CXCL1 expressions in colon tissues. **(C)** The relative intensities of CXCL1 as normalized against GAPDH (n = 4). Data are show as mean ± SEM. **P < 0.01, ***P < 0.001. To fit into the manuscript properly, the gel was reasonably trimmed.

**Figure 8:**
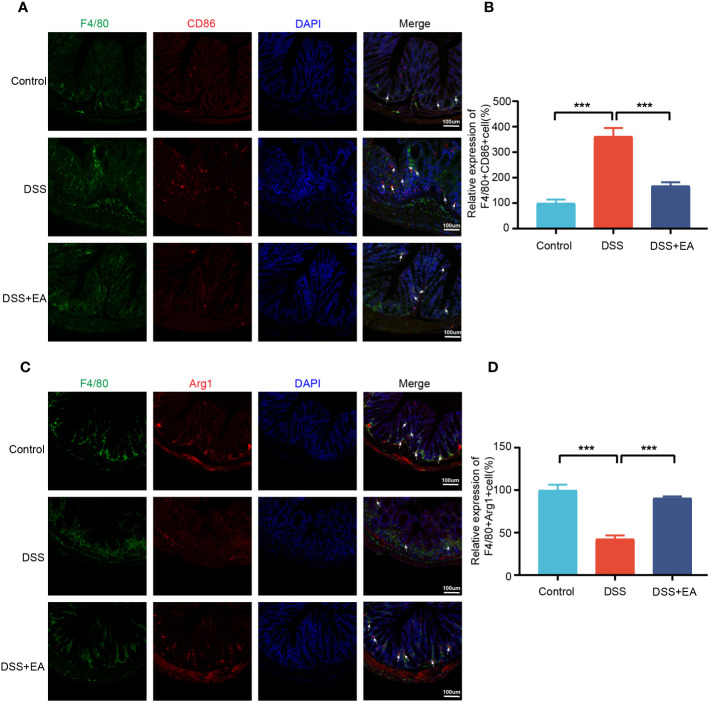
Colonic macrophage polarization in the different groups. **(A)** Colon macrophages M1 polarizations were stained with anti-F4/80 and anti-CD86 antibodies and observed by fluorescence image (green expressed F4/80+ cells, red expressed CD86+ cells, and blue expressed DAPI; the white arrow represents F4/80+CD86+ cells). **(B)** The relative expression of macrophage M1 polarization (F4/80+CD86+) in three groups (n=3). **(C)** Colon macrophages M2 polarizations were stained with anti-F4/80 and anti-Arg1 antibodies and observed by fluorescence image (green expressed F4/80+ cells, red expressed Arg1+ cells, and blue expressed DAPI; the white arrow represents F4/80+ Arg1+ cells). **(D)** The relative expression of macrophage M2 polarization (F4/80+Arg1+) in three groups (n = 3). Data are show as mean ± SEM. ***P < 0.001.

**Figure 9:**
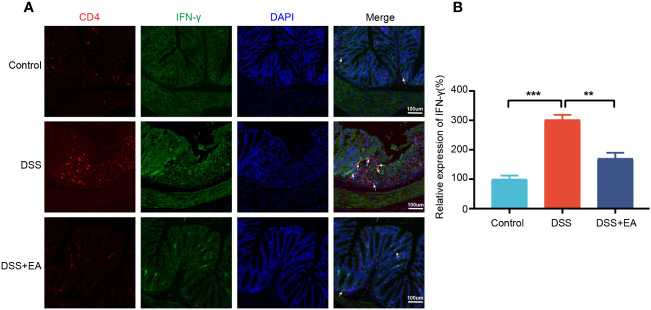
Colonic Th1 cytokine IFN-γ in the different groups. **(A)** Colon Th1 cytokine IFN-γ was stained with anti-CD4 and anti-IFN-γ antibodies and observed by fluorescence image (green expressed IFN-γ+ cells, red expressed CD4+ cells, and blue expressed DAPI; the white arrow represents CD4+ IFN-γ+ cells. **(B)** The relative expression of Th1 cytokine IFN-γ (CD4+ IFN+) in the three groups (n = 3). Data are show as mean ± SEM. **P < 0.01, ***P < 0.001.

By WB and IF, we founded that EA significantly reduced CXCL1 expression in UC mice (**Figures 7A–C**). But what pathway does EA affect the expression of CXCL1? As shown in the previous studies, CXCL1 was mainly secreted by activated macrophages. (27). Macrophages play a crucial role in the continuous renewal of intestinal epithelial cells and the maintenance of immune balance in the intestinal mucosa (28). Moreover, they exert a substantial impact on UC by modulating their phenotype polarization, either towards pro-inflammatory (M1) or anti-inflammatory (M2), in response to various environmental stimuli (28, 29). For this reason, we investigated the effects of EA on macrophage M1/M2 polarization. We gated M1 macrophages with markers F4/80, CD86 (F4/80+ CD86+ cells), and M2 macrophages with markers F4/80 and Arg1 (F4/80+ Arg-1+ cells) (**Figures 8A, C**). The results show that EA could promote M2 polarization of UC macrophages and inhibit M1 polarization (**Figures 8B, D**). Because macrophage polarization is induced by Th1 cytokine IFN-γ(30), we gated Th1 cytokine IFN-γ with markers CD4 and IFN-γ (CD4+ IFN-γ+ cells). The results showed that Th1 cytokine IFN-γ was increased after DSS modeling but was significantly reduced by EA treatment (**Figures 9A, B**), which is highly consistent with the results of human colon gene expression dataset immune infiltration analysis.

## Discussion

This study showed that EA is able to effectively improve the inflammatory cells infiltrations, hyperemia, edema, and ulceration of the colon in UC mice; and reduce the DAI score and the expression levels of serum TNF-α and IFN-γ as well as colonic IL-1β, IL-6, TNF-α, and IFN-γ. These findings indicate the positive role of EA in the treatment of UC. Since the genetic similarity between humans and mice makes mice a popular model to study human diseases (12), we next analyzed the transcription data of mice and compared it with human colon gene expression dataset GSE38713, so that the bioinformatics analysis results are more consistent with the clinical disease characteristics. Finally, eighteen co-DEGs were identified in joint bioinformatics analyses of mice and human transcriptional data, among which *Cxcl1* was the core gene. EA could affect IICs by inhibiting *Cxcl1* mRNA expression, and that EA regulated the polarization of macrophages by affecting the Th1 cytokine IFN-γ, thereby inhibiting the expression of CXCL1 immune response related pathways, such as ‘leukocyte migration,’ ‘secretory granule,’ ‘receptor ligand activity,’ and ‘IL-17 signaling pathway’, which were mainly enriched in both mice and human data.

According to previous studies, dysregulated genes in UC are primarily associated with immune function (31, 32), and ‘leukocyte migration,’ and ‘IL-17 signaling pathway’ contributes to inflammation and tissue damage in the colon (33, 34). In addition, EA has also been reported to modulate immune factors and promote colon recovery (35–37). Our functional annotation and pathway enrichment of RNA-seq data from mice and humans reveal that the immune process plays a crucial role in UC pathogenesis, and the mechanism of EA in treating UC may relate to regulating these immune processes.

Eighteen co-DEGs were identified by integrating homologous gene in mice and human transcriptome data. These co-DEGs were divided into two main categories: matrix metalloproteinases (*Mmp9*, *Mmp12*, and *Mmp3*) and the CXC family of chemokines (*Cxcl1*, *Cxcl3*, *Cxcl5*, and *Cxcl13*). Among them, *Cxcl1* was identified as the core gene by cytoHubba. To confirm the results of the bioinformatics analysis, the mRNA expression levels of *Cxcl1*, *Cxcl3*, *Cxcl5*, and *Cxcl13* were validated by qRT-PCR, which were consistent with the bioinformatics results. Chemokines may play an important role in the pathogenesis of UC, and recruit neutrophils into the gut and cause various inflammatory effects (38), including neutrophil activation, particle extravasation, and production of metalloproteinases to degrade the matrix (39, 40). Chemokines have also been implicated in the mechanism of UC from clinical and animal studies (41, 42) and there is evidence that chemokines can be used as biomarkers or targets to treat UC (43, 44). Such previous findings indicate that the CXC family of chemokines are essential targets in the regulation of UC by EA. To further clarify the correlation between *Cxcl1* and immune cells in UC, the ImmuCellAI database was used to calculate the infiltration fraction of 24 immune cells, and a correlation analysis confirmed that Cxcl1 correlated with Th1, Tfh, CD4+ T, nTreg, and CD4 naïve T cells.

*Cxcl1*, as a core gene closely related to immune cells in UC, is considered to be a key target for acupuncture treatment in inflammatory diseases (45, 46). Again, our research confirms this conclusion. CXCL1 is a chemokine that belongs to the CXC sub-family (27). In 2013, Katia De Filippo et al. (47) reported that macrophage chemokines CXCL1/CXCL2 control the early stage of neutrophil recruitment during tissue inflammation. Having arrived within the stimulated tissue, neutrophils penetrate further in a macrophage-dependent manner. In 2021, Moin et al. (48) found that the release of chemokines, including CXCL1 from activated M1 macrophages, is characteristic of the activation of basic M1 macrophages. Thus, it can be seen that macrophages have a close relationship with CXCL1. In the gastrointestinal mucosa, macrophages are widely distributed, mainly in the natural layer (LP) near the epithelium and in the smooth layer (SM) (49–51). Such distributional character indicates that macrophages play a vital role in the regulation of inflammation, the homeostasis of adipose tissue, and the defense of the host (51). It is further discovered that the imbalance of M1/M2 phenotypes of macrophages polarization has been shown to contribute to the exacerbation of colitis in the murine model of IBD (52). M1 macrophage phenotypes are characterized by secreting cytokines such as CD86, IL-1β, TNF-α, and ROS, inducing inflammation and clearing pathogens, and involved in the induction of T helper 1 cells (Th1) and Th17 responses (28, 53). In the inflamed gut’s lamina propria, M1 macrophages with pro-inflammatory properties degrade tight junction proteins, impair the epithelial barrier, and trigger apoptosis in epithelial cells, culminating in an overabundance of inflammation (54, 55). Conversely, M2 macrophages are commonly distinguished by the up-regulation of factors, including ARG1, IL-10, CD163, and CD206, which factors have the potential to impede excessive inflammation and facilitate tissue healing (28). It was identified that EA effectively mitigates DSS-induced colitis by modulating macrophage polarization through the suppression of NLRP3/IL-1β and the promotion of Nrf2/HO-1 (56). Additionally, previous studies have also confirmed that EA could effectively decrease the expression of IFN-γ and TLR4 (the factor that induced macrophages M1 polarization) while increase the expression level of IL-10 (the factor that induced macrophages M2 polarization) in the colon of DSS-induced mice (10, 56).

Our study proved that EA may involves in macrophage polarization in UC and EA favors M2 macrophage polarization while suppressing M1 macrophage polarization. We find that *Cxcl1* is closely related to Th1 cells by immune infiltration analysis. Furthermore, EA regulation of Th1 cytokine IFN-γ, a major macrophage activation factor responsible for M1 macrophage activation (57), was confirmed by IF detection. Our findings have not only identified *Cxcl1* as the core gene involved in the treatment of UC through EA but have also provided initial insights into the mechanism by which EA regulates the differentiation of Th1 cytokine IFN-γ, consequently influencing the polarization of macrophages and potentially impacting the immune mechanism underlying CXCL1 secretion.

## Conclusions

In conclusion, our study confirmed the positive effect of EA on UC. Further, it revealed that CXCL1 is the critical target for EA to take effect, and that the underlying immune mechanism is related to Th1 cytokine IFN-γ.

## Data availability statement

The datasets presented in this study can be found in online repositories. The names of the repository/repositories and accession number(s) can be found below: https://www.ncbi.nlm.nih.gov/geo/, GSE227407; https://www.ncbi.nlm.nih.gov/geo/, GSE38713.

## Ethics statement

The animal study was approved by Animal Experimental Ethics Committee of the Chengdu University of Traditional Chinese Medicine. The study was conducted in accordance with the local legislation and institutional requirements.

## Author contributions

R-BZ drafted the manuscript and analyzed the data. L-CD performed most of the experiments. R-BZ and L-CD performed animal breeding and part of the animal experiments. Q-FW and S-GY performed and supervised the study. YS and H-YL provided suggestions for this study and revised the article. All authors contributed to the article and approved the submitted version.
