# Supplementary material for: Electroacupuncture alleviates ulcerative colitis by targeting CXCL1: evidence from the transcriptome and validation
Source: Front Immunol. 2023 Sep 1;14:1187574. doi: 10.3389/fimmu.2023.1187574 (PMC10505654; doi:10.3389/fimmu.2023.1187574)
Supplement: Supplementary file 1 [file Table_1.docx]

| **Feature** | **Score** | **Description** |
| --- | --- | --- |
| Body weight loss | 0 | 0% |
|  | 1 | 1-5% |
|  | 2 | 6-10% |
|  | 3 | 11-15% |
|  | 4 | >15% |
| Feces status | 0 | Normal |
|  | 2 | Loose stools |
|  | 4 | Watery stool |
| Occult/Bloody stools | 0 | Normal |
|  | 2 | Hemoccult positive |
|  | 4 | Hematochezia with nakedeyes |

Supplementary Table 1 Sores of disease activity index (DAI)
