# Supplementary material for: Electroacupuncture alleviates ulcerative colitis by targeting CXCL1: evidence from the transcriptome and validation
Source: Front Immunol. 2023 Sep 1;14:1187574. doi: 10.3389/fimmu.2023.1187574 (PMC10505654; doi:10.3389/fimmu.2023.1187574)
Supplement: Supplementary file 2 [file Table_2.docx]

| Feature | Score | Description |
| --- | --- | --- |
| Inflammation | 0 | None |
|  | 1 | Minimal multifocal inflammation (few foci) |
|  | 2 | Moderate multifocal inflammation (numerous foci) |
|  | 3 | Severe multifocal coalescing inflammation |
| Extent | 0 | None |
|  | 1 | Mucosa |
|  | 2 | Mucosa and submucosa |
|  | 3 | Transmural |
| Crypt damage | 0 | None |
|  | 1 | Basal 1/3 damaged |
|  | 2 | Basal 2/3 damaged |
|  | 3 | Only surface epithelium intact |

Supplementary Table 2 Histological scores of colitis.
