# Supplementary material for: Electroacupuncture alleviates ulcerative colitis by targeting CXCL1: evidence from the transcriptome and validation
Source: Front Immunol. 2023 Sep 1;14:1187574. doi: 10.3389/fimmu.2023.1187574 (PMC10505654; doi:10.3389/fimmu.2023.1187574)
Supplement: Supplementary file 3 [file Table_3.docx]

| Primer name | Sequence (5' -> 3') |
| --- | --- |
| *Cxcl1-F* | CTGGGATTCACCTCAAGAACATC |
| *Cxcl1-R* | CAGGGTCAAGGCAAGCCTC |
| *Cxcl3-F* | CCAGACAGAAGTCATAGCCAC |
| *Cxcl3-R* | CGTTGGGATGGATCGCTTTTC |
| *Cxcl5-F* | TCCAGCTCGCCATTCATGC |
| *Cxcl5-R* | TTGCGGCTATGACTGAGGAAG |
| *Cxcl13-F* | GGCCACGGTATTCTGGAAGC |
| *Cxcl13-R* | GGGCGTAACTTGAATCCGATCTA |
| *β-actin-F*  *β-actin-R* | GGCTGTATTCCCCTCCATCG  CCAGTTGGTAACAATGCCATGT |

Supplementary Table 3 qPCR primer name and sequence
